# Neurochemical and Cognitive Beneficial Effects of Moderate Physical Activity and Catechin in Aged Rats

**DOI:** 10.3390/antiox10040621

**Published:** 2021-04-19

**Authors:** Margarita R. Ramis, Fiorella Sarubbo, David Moranta, Silvia Tejada, Jerònia Lladó, Antoni Miralles, Susana Esteban

**Affiliations:** 1 Laboratory of Neurophysiology, Biology Department, University of Balearic Islands (UIB), Ctra. Valldemossa Km 7.5, E-07122 Palma de Mallorca, Spain; margaramis87@gmail.com (M.R.R.); mariafiorella.sarubbo@hsll.es (F.S.); david.moranta@uib.es (D.M.); silvia.tejada@uib.es (S.T.); amiralles@uib.es (A.M.); 2 Research Unit, University Hospital Son Llàtzer, Crta. Manacor Km 4, 07198 Palma, Spain; 3 CIBERON (Physiopathology of Obesity and Nutrition), 28029 Madrid, Spain; 4 Health Research Institute of the Balearic Islands (IdISBa), 07120 Palma, Spain; 5 Department of Biology and University Institute of Health Sciences Research (IUNICS-IdISBa), University of Balearic Islands, 07122 Palma, Spain; jeronia.llado@uib.es

**Keywords:** physical activity, memory, monoamines, SIRT1, brain health, aging, catechin, 5-HT, Noradrenaline (NA), Dopamine (DA)

## Abstract

A healthy aging process is a requirement for good life quality. A relationship between physical activity, the consumption of antioxidants and brain health has been stablished via the activation of pathways that reduce the harmful effects of oxidative stress, by inducing enzymes such as SIRT1, which is a protector of brain function. We analyzed the cognitive and neurochemical effects of applying physical exercise in elderly rats, alone or in combination with the antioxidant catechin. Several tests of spatial and episodic memory and motor coordination were evaluated. In addition, brain monoaminergic neurotransmitters and SIRT1 protein levels were assessed in the brains of the same rats. The results show that physical activity by itself improved age-related memory and learning deficits, correlating with the restoration of brain monoaminergic neurotransmitters and SIRT1 protein levels in the hippocampus. The administration of the antioxidant catechin along with the exercise program enhanced further the monoaminergic pathways, but not the other parameters studied. These results agree with previous reports revealing a neuroprotective effect of physical activity, probably based on its ability to improve the redox status of the brain, demonstrating that exercise at an advanced age, combined with the consumption of antioxidants, could produce favorable effects in terms of brain health.

## 1. Introduction

Aging is a normal physiological process characterized by a general decline in brain functions, including cognitive and motor capacity [1]. Due to the increase in life expectancy, the prevention of this process have become one of the most outstanding research challenges to address in the coming years, since it may compromise the ability of the elderly to maintain an active and independent lifestyle. Therefore, it also has an important impact on society and it is crucial to know more about possible feasible preventive therapies that could mitigate the normal aging process.

Physical exercise is one of the components of lifestyle that has generated a great deal of interest in recent years, as it seems to have a beneficial impact on health, especially on cognition and mood [2,3,4]. Studies focusing on this topic have determined the effects of moderate exercise on brain function as positive, frequently without elucidating the specific cause of these effects. Therefore, mechanisms involved in the beneficial effects of exercise should be addressed from different points of view, beginning with the molecular processes mainly involved in aging. In this sense, at a molecular level, the decline in cognitive functions during normal aging has been found to be correlated with brain oxidative stress [5] and neuroinflammation [6], which influence each other, affecting the correct functionality of the brain. It has been disclosed that physical activity elicits a combined effect, improving the redox state and enhancing inflammatory defenses in aged subjects [7,8]. A number of investigations have also suggested that free radicals can act as modulators of several signaling pathways linked to the activation of metabolic sensors of cellular redox state, such as the sirtuins. Among them, SIRT1 (Sirtuin1) and SIRT3 (Sirtuin 3) are NAD-dependent histone deacetylase enzymes that regulate a wide variety of cellular responses such as cell survival, apoptosis and mitochondrial biogenesis [9,10]. It has been suggested that SIRT1 improves cell survival in front of harmful signals throughout aging by modulating pathways involved in cell protection, such as those related to inflammation or oxidative stress [11]. In this way, SIRT1 has been shown to induce the antioxidant protein MnSOD and to reduce the activation of proinflammatory cytokines via the downregulation of NF-κB [12]. These antioxidant effects are mediated at least in part by FOXO transcription factors [13,14]. The role of SIRT1 in brain aging includes the modulation of synaptic plasticity and cognition [15]. Importantly, there was a decrease demonstrated in SIRT1 levels in the hippocampus of aged rats, which was reverted by chronic treatment with antioxidants such as polyphenols [16,17]. Another protein expressed in the hippocampus associated with age-related memory loss is RbAp (histone-binding protein RbAp48/retinoblastoma-binding protein), which also modulates histone acetylation patterns similar to SIRT1 [18,19].

Furthermore, it is well known that these alterations observed in aging, in both animals and humans, are also associated with changes in brain regions, especially in the hippocampus [20], a brain area important for cognition and memory processes.

It is well known that exercise can enhance the favorable effects of a healthy diet and vice versa. Some studies described that certain polyphenols improved the positive effects of exercise on cognitive functions [21]. In a previous study we demonstrated the beneficial effects of catechin, a flavanol present in several foods [17] largely consumed worldwide, on brain function.

Therefore, the present work aims to evaluate the effect of moderate physical activity and also the possible additional action of the antioxidant catechin, a polyphenol found in the diet, on cognitive functions, by using hippocampus-dependent behavioral tests in aged rats. Our hypothesis is that physical exercise together with catechin could protect the cognitive functions and brain plasticity of aged animals by modulation of: (1) the synthesis and metabolism of monoamines (5-HT, NA and DA) involved in the memory process in several regions including the hippocampus; and (2) hippocampal SIRT1 and RbAp proteins, which regulate normal brain function and are particularly associated with age-related memory loss.

## 2. Materials and Methods

### 2.1. Animals and Treatments

Male 18-month-old Wistar rats were included in the experiment (*n* = 21; 600 ± 35 g, Charles River, Barcelona, Spain). Environmental conditions (20 ± 2 °C; 70% humidity, and 12:12 light:dark (LD) cycle, lights on at 08.00 h) were controlled to house animals individually with water and standard laboratory animal food (Panlab A04, Barcelona, Spain) ad libitum. Control group (*n* = 7) received corn oil (1 mL/kg/day, i.p.). For 5 weeks, one group of rats (*n* = 7) did exercise for 5 days/week (Monday to Friday) in the rotarod device. Based on the time and speed that older animals could easily stand on the rotarod, we designed a protocol in which both the time and the speed were progressively increased so the animals could adapt to this exercise. The first 5 days, rats were set for 5 min in the wheel while speed increased from 4 to 10 rpm; the next two weeks, the speed was increased from 10 to 15 rpm while animals were also maintained for 5 min in the device; and for the last two weeks the speed was constant at 15 rpm, but the animals remained on the rotating wheel for 10 min. This timetable allowed a gradual increase in the duration and intensity of the physical exercise as the animals improved their performance. The second group of animals (*n* = 7) completed the same physical exercise procedure described in the previous group, and for the first 28 days they also received 20 mg/kg/day of the antioxidant catechin (diluted in corn oil, 1 mL/kg/day, i.p.) and 40 mg/kg/day (i.p.) for the following 7 days, following the same pattern as in a previous work in which it showed favorable effects on cognitive and neurochemical abilities in old rats [17]. Both the control group (*n* = 7) and exercise group received corn oil (1 mL/kg/day, i.p.) during the 35 days of treatment. The same rats were studied throughout the study. Cognitive abilities were checked along the chronic treatment (35 days) (see next sections). When the behavioral analysis was completed, animals were sacrificed by decapitation at the end of the treatments.

A single injection of NSD 1015 (100 mg/kg, i.p., central aromatic amino acid decarboxylase inhibitor) was administered to all rats 30 min before the sacrifice so as to analyze the in vivo activity of limiting enzymes in monoamine synthesis; then the tryptophan hydroxylase (TPH) and tyrosine hydroxylase (TH) activities were measured by the accumulation of 5-hydroxytryptophan (5-HTP) and dihydroxyphenylalanine (DOPA), respectively, over 30 min (see below). Hippocampus, striatum (caudate-putamen) and pineal gland were quickly dissected from brains on an ice-cold plate; then the tissues were frozen in liquid nitrogen immediately and stored at −80 °C until analysis.

All procedures were performed in accordance with the EU Directive 2010/63/EU of the European Parliament and of the Council, following the Spanish Royal Decree 53/2013 and the Local Bioethical Committee (University of the Balearic Islands), and were approved by the Bioethical Committee of the University (approval file number 2014/05/AEXP).

### 2.2. Behavioral Tests

Different behavioral tests dependent on hippocampal and striatal function were used. Half an hour before the tests, rats were familiarized with the experimental room and devices. Each animal was evaluated individually between 9:00 and 13:00 h, rotating the order of the different groups to prevent possible chronobiological effects. All the devices used to assess cognition were cleaned with ethanol 90% before each test.

#### 2.2.1. Motor Coordination in Rotarod

To assess the motor ability and balance of the animals, the rotarod treadmill device (Panlab^®^, Barcelona, Spain) was used. Four training sessions, one per day, were done on the rotating wheel in order to familiarize rats with the device. For the test phase, rats were placed on the rotarod to record the latency to fall in an acceleration mode (from 4 to 40 rpm over a period of 60 s). Five trials were performed by each rat, separated by a few minutes for the animal’s physical recovery, and competency was defined as the average time recorded.

#### 2.2.2. Spatial Working Memory in Radial Maze Test

Spatial working memory in rats was evaluated by the 8-arm radial maze (Panlab^®^) as previously reported [22]. It consisted of 8 arms (50 cm long, 12 cm wide) equidistantly separated and placed around an octagonal central platform (30 cm diameter) which was the entrance in each arm. At the end of each arm, food pellets were allocated as positive stimulus for the exploration of the maze. Additionally, for better testing of spatial memory, there were allocated cues of different forms, colors and shapes on the room walls where the maze was placed. To achieve a convenient motivational level, rats were previously submitted to 48 h fasting [23]. Radial maze was performed every 15 days throughout the treatment. To assess working memory, rats had to enter each arm to obtain small pieces of food pellets until all eight arms had been visited or 20 min had elapsed in the trial. The sum of non-visited arms and re-entry into arms were considered as errors. Movements of the animals and the distance traveled were monitored by a digital video tracking system (LE 8300 with software SEDACOM v 1.3, Panlab^®^) which was analyzed with the software SMART v 2.5 (Panlab^®^).

#### 2.2.3. Visuospatial Learning in Barnes Holeboard Maze

The maze consisted of a 130 cm circular disk with 18 holes located around the perimeter equidistantly at 75 cm high. A black box or target (30 cm long, 20 cm wide, 10 cm height) was located under one of the holes, which was indistinguishable from the other ones from the center of the maze. The experimental room had different visual cues as reference points to localize the target (scape box). As a stimulus to find the target, a bright light was turned on to accentuate the natural agoraphobia of rats [24]. The test was carried out at the end of the treatments, and a familiarization phase was done the day before in which animals were allowed to explore the maze freely in order to be familiarized with the device. The rat was placed in the center of the maze, the light was turned on, and 3 min were given to explore the maze and find the box. Rats that did not find the box were placed manually in the scape box during 1 min. When the animal was inside the box, the light was turned off. The test consisted of the conduction of four trials every ten minutes. The trial was considered done when the rat reached the target, or after 3 min when animals were placed into the target box manually and remained there for 1 min. Latency was defined as the time used to explore the maze until attaining the target, and an error as the exploration of the non-target holes. Three different strategies to search the target were considered: direct (animals enter directly the target), serial (animals explore holes sequentially) or random (other patterns to reach the target), as described by Rueda-Orozco and cols [25].

#### 2.2.4. Non-Spatial Memory in Novel Object Recognition Test

New object recognition is a test to quantify episodic-like memory in rodents; it assesses the natural tendency of the rats to explore based on the spontaneous exploratory activity novelty [26]. An open field device (78 cm diameter cylinder with a 60 cm high opaque wall) was used for habituation, familiarization and the test phase [17,27]. During the habituation phase each animal explored the open field freely for 10 min without objects, once per day for 4 consecutive days. Re-habituation was done on the fifth day, in which rats were allocated 1 min in the device for the recognition of the place. In the next phase, familiarization, rodents were set in the center of the platform with two identical objects (e.g., plastic blue cubes 6 cm long, 6 cm wide, 6 cm height) always placed in the same position (16 cm away from the walls, 35 cm between them). Rats were allowed to explore objects for up to a maximum of 10 min, considering a direct contact with the object with the nose or mouth an “object exploration”. The test phase was carried out 10 min later, during which the animals were repositioned in the open field with one familiar object (one used in the previous phase but previously cleaned) and one new object (e.g., a plastic red bolus 8 × 4 cm). The time used to explore the objects was recorded for 10 min. The objects were made with the same material, although with dissimilar shapes, and they had never been related to reinforcement or location, avoiding natural significance for the animals. The time spent with both objects in this phase reflected the preference for novelty.

Discrimination index (D.I.) was calculated following the equation:

D.I. = (time exploring novel object − time exploring familiar object)/total time exploring any object.

The whole device and objects were cleaned using ethanol 90% in order to avoid clues between trials. Furthermore, the number of times that the rats passed through the different areas of the open field and the number of urinations and defecations were recorded so as to measure anxiety and exploratory drive of the rats.

### 2.3. Neurochemical Analysis

#### 2.3.1. Tryptophan Hydroxylase (TPH) and Tyrosine Hydroxylase (TH) Activity

In the current work, to assess the in vivo synthesis of 5-HT, DA and NA, the activity of tryptophan hydroxylase (TPH, tryptophan-5-monoxygenase, EC 1.14.16.4)—as the rate-limiting enzyme for 5-HT synthesis—and tyrosine hydroxylase (TH, tyrosine-3-monoxygenase, EC 1.14.16.2)—as the rate-limiting enzyme for DA and NA synthesis—were measured by determining the 5-HTP and DOPA accumulation within 30 min after inhibition of the aromatic L-amino acid decarboxylase (EC 4.1.1.28) by a maximally effective dose of NSD-1015 (100 mg/kg, i.p.) [22]. The 5-HTP and DOPA accumulation method is the main biochemical assay used to measure the in vivo rate of tryptophan and tyrosine hydroxylation in the brain. Moreover, the pool of 5-HT, DA and NA unaffected by new synthesis primarily stored neurotransmitter intraneuronally, and the metabolite levels let us analyze the recent use of these neurotransmitters. TPH occurs in two isoforms: TPH-1 is mainly expressed in the pineal gland and in gut enterochromaffin cells [28], while TPH-2 is preferentially expressed in the brain, where it plays a fundamental role in 5-HT synthesis [29]. High-performance liquid chromatography (HPLC; Waters, Barcelona, Spain) with electrochemical detection was used to assess the 5-HTP and DOPA formed from endogenous tryptophan and tyrosine, respectively, and the other compounds in the hippocampus (5-HT and NA terminal-rich region) and corpus (dorsal) striatum (rich in 5-HT and DA nerve terminals). The activity of the TPH-2 isoform is responsible for the 5-HTP accumulation in the hippocampus and striatum; while the 5-HTP formed in the pineal gland is mediated by TPH-1. Tyrosine hydroxylation is a common step in the synthesis of catecholamines, so the DOPA accumulation in the striatum preferentially indicates DA synthesis, and accumulation in the hippocampus is mainly related to the NA synthesis. The output electric current was monitored by an interphase (Waters bus SAT/IN Module) connected to a computer. The concentrations of the compounds in a given sample were calculated by interpolating the corresponding peak height into a parallel standard curve using the software Empower Pro (Waters). See Ramis and cols. [17,30] for more procedure details.

#### 2.3.2. SIRT1 and RbAp Proteins by Western Blot Analysis

The remaining parts of the rat hippocampus were homogenized in a 1:15 weight/volume ratio of cold homogenization buffer (50 mMTris-HCl, pH 7.5; 1 mM EDTA; 2% SDS) in the presence of a protease inhibitor cocktail (Pierce) with an Ultra-Turrax homogenizer (Type Tp 18/10, Janke and Kunkel, Staufen, Germany) two times for 10 s each sample. Then, extracts were also sonicated two times for 10 s. Total protein content from homogenates was analyzed using the bicinchoninic acid method following manufacturer’s instructions (PierceTM BCA Protein Assay Kit) and total protein content was adjusted to 6 µg/µl in each sample. Later, homogenates were mixed 1:1 with loading Laëmmli buffer. Protein samples (40 μg) were separated by 10% SDS-PAGE and transferred to nitrocellulose membranes (3 MM Whatman). Immunoblot analyses were performed by using the antibodies: anti-SIRT1 (rabbit polyclonal, 1:1000 dilution, Merck Millipore; Cat. #07-131); anti-RBAP48/46 (rabbit polyclonal, 1:1000 dilution, Cell Signaling; Cat. 4633S); and anti-β-actin (mouse monoclonal, 1:10,000 dilutions, Sigma-Aldrich; Cat. A1978). Secondary HRP-linked antibodies consisted of anti-rabbit IgG (goat polyclonal, 1:5000 dilutions, Cell Signaling; Cat. #7074) and anti-mouse IgG (horse polyclonal, 1:5000 dilutions, Cell Signaling; Cat. #7076). Proteins were detected using the ECL Western Blotting Detection Reagents (Amersham). The chemiluminescence bands were detected by exposure to photographic films (HyperfilmAmersham) and digitalized with a GS-800 scanner, and the integrated optic density was analyzed with the QuantityOne software (Bio-Rad). Every sample was analyzed no less than 3 times in different gels and membranes were reproved for β-actin, which was used for protein normalization. Unless otherwise stated, the other reagents were purchased from Sigma-Aldrich (Merck group, Darmstadt, Germany).

### 2.4. Statistics

Two-way or three-way ANOVA were applied for statistical analysis of behavioral outcomes using the Bonferroni or Fisher post-hoc test for pairwise statistical comparisons; two-way repeated measures ANOVA was used to compare the same animals throughout the treatment. One-way ANOVA and Student t-test were used for neurochemical evaluations. Results are expressed as mean ± S.E.M. and *p* ≤ 0.05 was considered statistically significant. Graph-Pad Prism (version 6.0) was used to analyze the data.

## 3. Results

### 3.1. Body Weight and Motor Coordination

**Figure 1:**
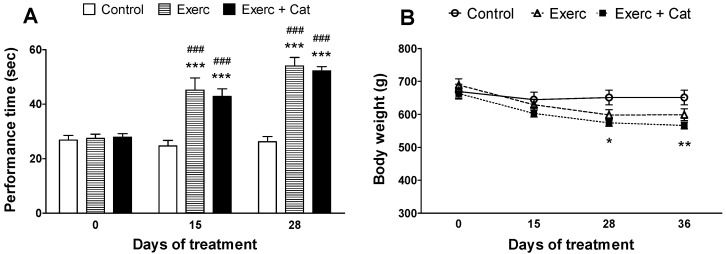
Effect of exercise and the combined action of exercise and catechin treatment on motor coordination (**A**) and the evolution of body weight (**B**) in old rats (18 months). (**A**) Bars represent the mean ± SEM of the time spent (sec) on the rotating wheel, using the rotarod test, of animals subjected to a daily physical exercise program (Exerc, *n* = 7) and animals subjected to the combined effect of a daily physical exercise program and intraperitoneal administration of catechin (20 mg/kg/day, *n* = 7; Exerc + Cat,) compared to control animals (*n* = 7). Two-way repeated measures ANOVA detected a significant effect for the treatment (F(2,54) = 19.6, *p* < 0.0001), the subjects (F(18,54) = 4.6, *p* < 0.0001), the age (F(2,54) = 78, *p* < 0.0001) and the interaction (F(4,54) = 21.90, *p* < 0.0001). B) Dots represent the mean ± SEM of the body weight (g) of the same animals. Two-way repeated measures ANOVA detected a significant effect for the subjects (F(18,72) = 33.12, *p* < 0.0001), the age (F(3,72) = 88.50, *p* < 0.0001) and the interaction (F(6,72) = 12.46, *p* < 0.0001), but not for the treatment (F(2,72) = 2.451, *p* = 0.1145). * *p* < 0.05, ** *p* < 0.01, *** *p* < 0.001 when compared to the control group; ### *p* < 0.001 comparing the values obtained by each group before and after performing the treatments (two-way ANOVA followed by Bonferroni).

Figure 1A shows the permanence time (sec) in the rotarod apparatus. The results indicate that after 15 days of exercise (alone or in combination with catechin), a significant increase in motor coordination was observed in the animals subjected to moderate physical activity, compared to the control group. These beneficial effects were also observed at 28 days of treatment (Exercise: increase of 105%, *p* < 0.001; Exercise + Catechin: 98%, *p* < 0.001, compared to control).

Likewise, a slight weight loss was observed throughout the study (Figure 1B) in the two groups that performed exercise (Exercise: 8%, Exercise + Catechin: 12%, compared to control), which could favor the execution of the test. No weight changes were detected in the control group.

### 3.2. Cognitive Abilities of Rats Along the Chronic Treatment

**Figure 2:**
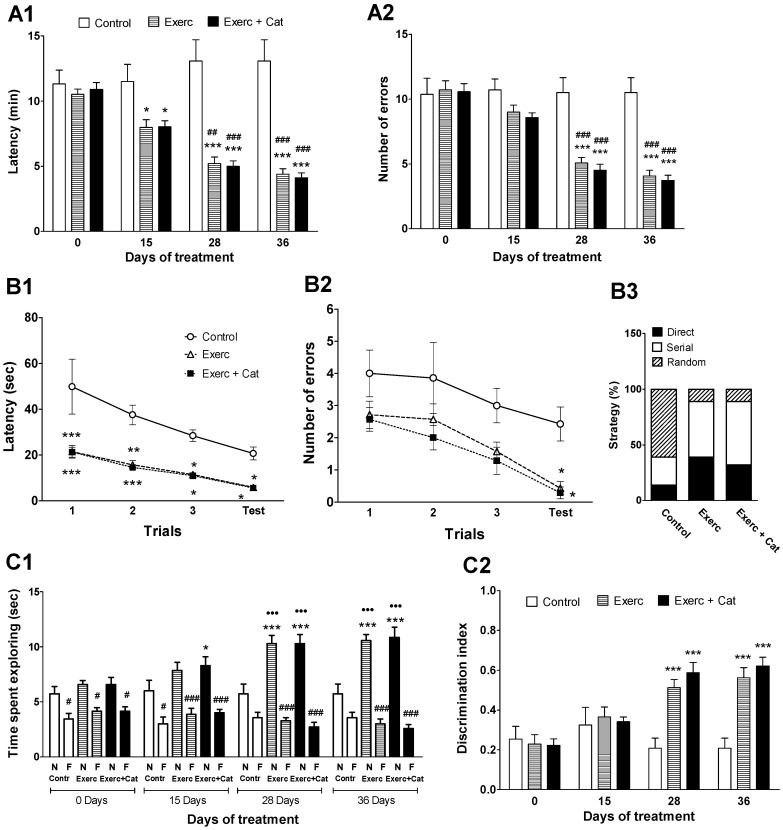
Effects of exercise and the combined action of exercise and catechin on behavioral tests. (**A**) Effect on spatial working memory using the radial maze test in old rats. Bars represent the mean ± SEM of time taken to complete the test (**A1**) and errors made (**A2**) of animals subjected to exercise (Exerc, *n* = 7) and exercise together with catechin antioxidant treatment (Exerc + Cat, *n* = 7) versus the control group (*n* = 7). (**A1**) Two-way repeated measures ANOVA detected a significant effect of treatment (F(2,72) = 14.6, *p* = 0.0002), age (F(3,72) = 30.5, *p* < 0.0001), subjects (F(18,72) = 9.26, *p* < 0.0001) and the interaction (F(6,72) = 18.5, *p* < 0.0001). (**A2**) Two-way repeated measures ANOVA detected a significant effect of treatment (F(2,72) = 10.1, *p* = 0.0012), age (F(3,72) = 60.9, *p* < 0.0001), subjects (F(18,72) = 7.22, *p* < 0.0001) and the interaction (F(6,72) = 15.5, *p* < 0.0001). (**B**) Effect on visuospatial learning ability using the Barnes maze test in old rats. Dots represent the mean ± SEM of latency (**B1**) and errors made (**B2**) throughout the Barnes test of the same groups as A. The bars represent the average of the strategy followed in % with respect to the total sum of the 3 training and test stages (**B3**) of the animals. (**B1**) Two-way ANOVA repeated measures detected a significant effect of treatment (F(2,72) = 21.3, *p* < 0.0001), trial (F(3,72) = 17, *p* < 0.0001) and subjects (F(18,72) = 2.09, *p* = 0.0191), but not for the interaction (F(6,72) = 0.938, *p* = 0.4759). (**B2**) Two-way ANOVA repeated measures detected a significant effect of treatment (F(2,72) = 10.6, *p* = 0.0009) and trial F(3,72) = 10.1, *p* < 0.0001), but not for the subjects (F(6,72) = 1.38, *p* = 0.1820) or the interaction (F(6,72) = 0.161, *p* = 0.9859). ** *p* < 0.001 comparing with the control group; ## *p* < 0.01, ### *p* < 0.001 comparing the values obtained before and after performing the treatments in each group (two-way ANOVA, followed by Bonferroni test). (**C**) Effect on episodic memory using the novel object recognition test in old rats. Bars represent the mean ± SEM of the total time spent exploring for each object (N, novel object; F, familiar object) in the test phase (**C1**), as well as the discrimination index between the novel and familiar object in the test phase (**C2**) of same groups of animals. (**C1**) Three-way ANOVA detected a significant effect of treatment (F(2,144) = 17.179, *p* = 0.0327), age (F(3,144) = 2.999, *p* < 0.0001) and objects (F(1,144) = 296.749, *p* < 0.0001). (**C2**) Two-way ANOVA repeated measures detected a significant effect of treatment (F(2,72) = 8.77, *p* = 0.0022), age (F(3,72) = 16.8, *p* < 0.0001), subjects (F(18,72) = 2.68, *p* = 0.0027) and interaction (F(6,72) = 8.14, *p* < 0.0001). * *p* < 0.05, *** *p* < 0.001 when compared to the control group; # *p* < 0.05, ### *p* < 0.001 comparing the exploration time between the novel object and the familiar object; ●●● *p* < 0.001 when comparing the values obtained before and after performing the treatments in each group (**C1**: three-way ANOVA, followed by Fisher PLSD test; **C2**: two-way ANOVA, followed by Bonferroni test).

A clear improvement in spatial memory as a result of the physical activity and the combined action of exercise and catechin treatment was observed. Exercise induced an improvement in the performance of the radial maze test (Figure 2(A1,A2)) after 15 days of the treatment, decreasing the time required to complete the test and the errors made. At the end, the treatments decreased the time required to finish the test (Exercise: 66%, *p* < 0.001; Exercise + Catechin: 69%, *p* < 0.001) compared to the control. Animals subjected to exercise made significantly fewer errors (61%, *p* < 0.001), as did animals in the Exercise + Catechin group (65%, *p* < 0.001), compared to the control group.

Spatial learning was evaluated at the end of the treatment with the Barnes maze test. All animals learned to locate the target throughout the training sessions but the animals submitted to physical activity performed better than controls (Figure 2(B1,B2)). In the first trials, animals submitted to physical activity remembered the target location learned the previous day (familiarization phase) better than control animals. Exercise significantly reduced latency and errors, with no changes observed with catechin administration. In addition, it could be observed that these animals mainly followed the serial and direct strategies to locate the escape box, whereas the controls mostly followed the random (Figure 2(C3)).

A positive effect of the exercise was also observed in the open field device. In the familiarization phase of the new object recognition test (Figure S1), the animals in the different groups did not show a preference for either object, although at the end of the experiment the animals in the Exercise and Exercise + Catechin groups explored the objects for a longer time (93% and 91%, respectively) compared to the control group. However, the discrimination of objects reached the level of statistical significance in the test phase (Figure 2(C1,C2)). At the end of the experiment, animals in the Exercise group explored the novel object 85% more and the familiar object 16% less compared to controls, while such exploration in the case of the Exercise + Catechin group was 90% more for the novel object and 28% less for the familiar object.

### 3.3. Effect of Exercise on Monoamine Synthesis and Metabolism in Hippocampus, Striatum and Pineal Gland

**Figure 3:**
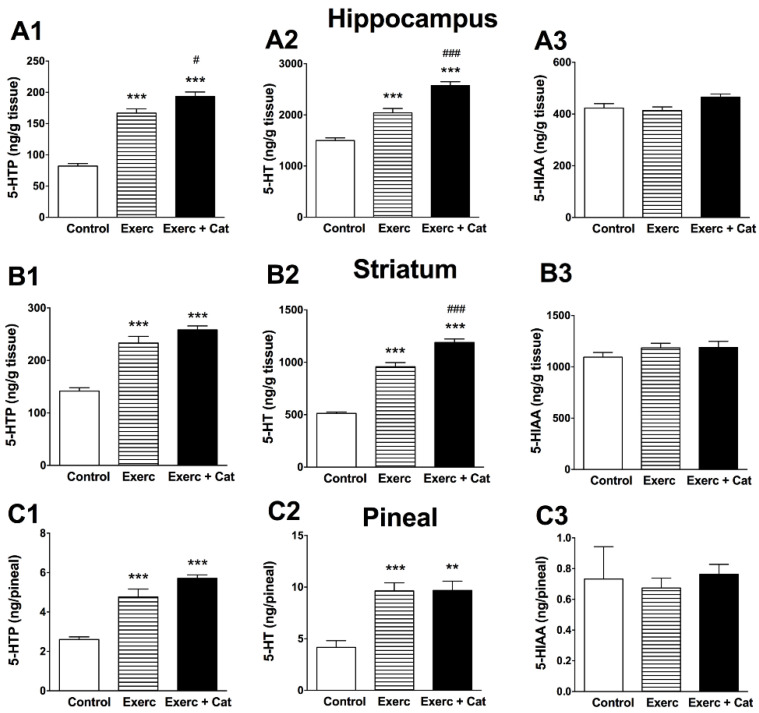
Effect of exercise and the combined action of exercise and catechin treatment on TPH-2 enzyme activity in the hippocampus (**A**) and striatum (**B**), and the effect on TPH-1 enzyme activity in the pineal gland (**C**) of aged rats. Bars represent the mean ± SEM of 5-HTP accumulation (after inhibition of decarboxylation with NSD-1015 for 30 min) (**A1**,**B1**,**C1**), 5-HT levels (**A2**,**B2**,**C2**) and 5-HIAA metabolite (**A3**,**B3**,**C3**) in extracts from the hippocampus and striatum (ng/g) and ng in the entire pineal gland (ng/pineal) of animals subjected to exercise (Exerc, *n* = 7) and the exercise plus catechin treatment group (Exerc + Cat, *n* = 7) versus control group animals (*n* = 7). (**A**) One-way ANOVA detected a significant effect on 5-HTP (F(2,18) = 100.2, *p* < 0.0001), 5-HT (F(2,18) = 61.74, *p* < 0.0001) and 5-HIAA (F(2,18) = 3.425, *p* = 0.05) in the hippocampus. (**B**) One-way ANOVA detected a significant effect on 5-HTP (F(2,18) = 141.2, *p* < 0.0001) and 5-HT (F(2,18) = 48.73, *p* < 0.0001), but not 5-HIAA (F(2,18) = 1.232, *p* = 0.314) in the striatum. (**C**) One-way ANOVA detected a significant effect on 5-HTP (F(2,16) = 23.6, *p* < 0.0001) and 5-HT (F(2,16) = 14.9, *p* = 0.0003), but not 5-HIAA (F(2,16) = 0.153, *p* = 0.859) in the pineal gland. ** *p* < 0.01, *** *p* < 0.001 comparing with the control group; # *p* < 0.05, ### *p* < 0.001 comparing both exercise groups with each other (one-way ANOVA, followed by Bonferroni test).

**Figure 4:**
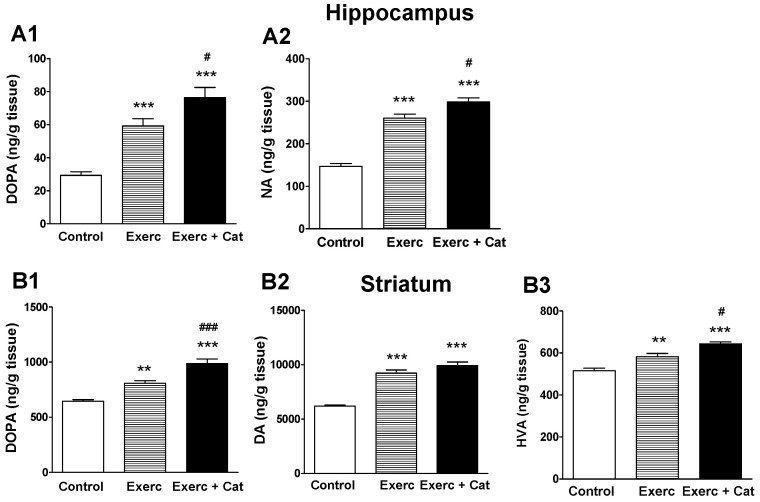
Effect of exercise and the combined action of exercise and catechin treatment on TH enzyme activity in the hippocampus (**A**) and striatum (**B**) of old rats. Bars represent the mean ± SEM of DOPA accumulation (after in vivo inhibition of decarboxylation with NSD-1015) (**A1**) and NA levels (**A2**) in extracts from the hippocampus, DOPA accumulation (**B1**) and DA levels (**B2**) as well as its metabolite HVA (**B3**) in extracts from the striatum of animals subjected to exercise (Exerc, *n* = 7) and the exercise plus catechin treatment group (Exerc + Cat, *n* = 7) versus animals in the control group (*n* = 7). (**A**) One-way ANOVA detected a significant effect on DOPA (F(2,18) = 30.51, *p* < 0.0001) and NA (F(2,18) = 88.83, *p* < 0.0001) in the hippocampus. (**B**) One-way ANOVA detected a significant effect on DOPA (F(2,18)=41.41, *p* < 0.0001), DA (F(2,18) = 63.07, *p* < 0.0001) and HVA (F(2,18) = 25.32, *p* < 0.0001) in the striatum. ** *p* < 0.01, *** *p* < 0.001 comparing with the control group; # *p* < 0.05, ### *p* < 0.001 comparing both exercise groups with each other (one-way ANOVA, followed by Bonferroni).

A positive effect of exercise was observed on TPH and TH activity (limiting enzymes in the synthesis of 5-HT, NA and DA) in brain regions critical in cognitive functions such as the hippocampus and striatum, and also in the pineal gland (Figure 3 and Figure 4).

TPH-2 enzyme activity in the hippocampus (Figure 3A) and striatum (Figure 3B) increased as a result of exercise, and an additional effect of catechin treatment was observed. In the hippocampus, an increase in 5-HTP levels was observed in the animals submitted to exercise (103%, *p* < 0.001) and even more in the animals submitted to exercise plus catechin (136%, *p* < 0.001), leading to an increase in 5-HT (36% and 72%, respectively, *p* < 0.001), with no significant alteration of the 5-HIAA metabolite. Similarly, in the striatum, the results show a significant modification of TPH-2 activity in the two groups of animals that underwent exercise. 5-HTP values showed a significant increase after both treatments (Exercise: 65%, *p* < 0.001; Exercise + Catechin: 83%, *p* < 0.001), leading to an increase in 5-HT (87% and 132%, respectively, *p* < 0.001) compared to the control group, with no changes in the 5-HIAA metabolite.

The synthesis of 5-HT was clearly modified in the pineal gland due to an increase in TPH-1 activity as a result of exercise (Figure 3C), but unlike TPH-2, no additional effect of catechin treatment was observed. As a consequence, 5-HTP increased significantly in the exercise group (83%, *p* < 0.001) and the exercise plus catechin treated group (119%, *p* < 0.001), and 5-HT levels also increased in both groups (131%, *p* < 0.001; 132%, *p* < 0.01, respectively) compared to control rats, without changes in 5-HIAA. These results obtained in the pineal gland of the animals submitted to exercise, compared with controls, suggest that physical activity can help restore pineal function by preventing the nocturnal decline of 5-HT (a precursor in the biosynthesis of melatonin, mediated by TPH-1) that normally occurs in the pineal gland as a result of aging [31].

The synthesis of NA mediated by TH was measured in the hippocampus (Figure 4A), a region rich in noradrenergic terminals. In this region, exercise produced a significant increase in DOPA levels (102%, *p* < 0.001) compared to the control group, an increase that was even greater in the group of animals that also received catechin (161%, *p* < 0.001). This increased availability of DOPA translated into an increase in the amount of NA in both groups (77% and 103%, respectively, *p* < 0.001). The synthesis of DA mediated by TH was measured in the striatum (Figure 4C), a brain region rich in dopaminergic nerve terminals. In the striatum, exercise also produced a significant increase in DOPA (Exercise: 25%, *p* < 0.01; Exercise + Catechin: 53%, *p* < 0.001), as well as an increase in DA (Exercise: 49%, *p* < 0. 001; Exercise + Catechin: 60%, *p* < 0.001) and its metabolite HVA (Exercise: 13%, *p* < 0.01; Exercise + Catechin: 25%, *p* < 0.001) compared to controls.

### 3.4. Effect of Exercise on SIRT1 Immunoreactivity in Hippocampus

**Figure 5:**
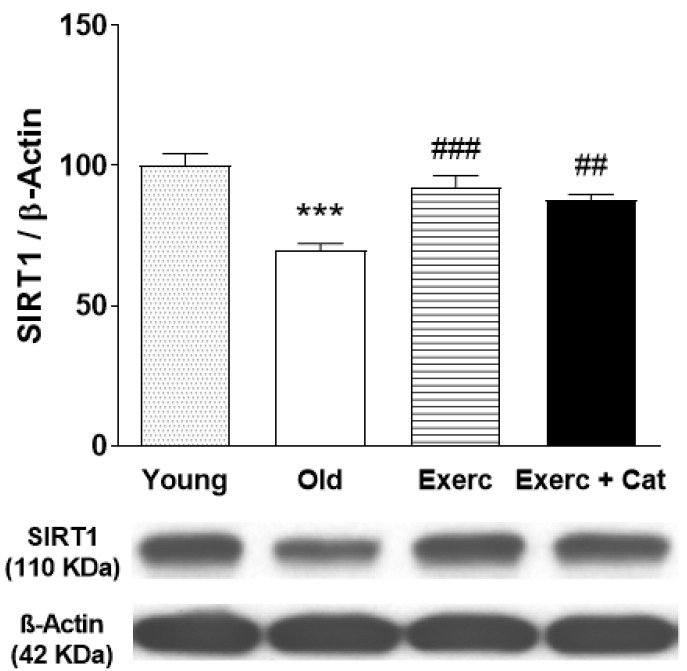
Effect of exercise and the combined action of exercise and catechin treatment on SIRT1 (110 kDa) protein expression in the hippocampus of old rats and young control rats. Bars represent mean ± SEM of protein levels per group (animals subjected to exercise, Exerc, *n* = 7; exercise together with catechin, Exerc + Cat, *n* = 7; old control group, Old, *n* = 6) expressed as a percentage with respect to the young control group (*n* = 6). Protein level was normalized to β-actin content and each sample was analyzed in three different membranes. One-way ANOVA detected a significant effect (F(3,22) = 17.48, *p* < 0.0001). *** *p* < 0.001 when compared with young animals in the control group; ## *p* < 0.01, ### *p* < 0.001 when compared with old animals in the control group (one-way ANOVA, followed by Bonferroni test). A representative immunoblot of the changes obtained in SIRT1, with no changes in ß-actin (loading pattern), is shown below the graph.

At the end of both exercise programs, an increase was observed in the hippocampal levels of SIRT1 protein. The immunoblot included a group of young rats to check the known negative effect of age on SIRT1 levels in correlation with the age-related decline in cognitive functions. Figure 5 shows the results obtained from the animals submitted to exercise show a significant increase in SIRT1 levels with respect to the old control animals (32%, *p* < 0.001). Similar results were obtained by the animals that practiced exercise and received catechin (26%, *p* < 0.01). The current results show that physical exercise can restore SIRT levels reduced with aging. In contrast, the possible role of RBAP46/48 in the protective effects of exercise was investigated (Figure S2), but the age-associated reduction in hippocampal RBAP protein levels observed in old animals was unchanged by the effect of physical exercise, indicating that RBAP46/48 seemed not to have a relevant role in the neuroprotective effects of exercise or catechin.

## 4. Discussion

Age-related cognitive decline has been linked to physical inactivity [32], so exercise could exert a protective effect on cognitive and motor abilities [33,34,35,36]. Weekly moderate exercise started at young age in mice decreased oxidative stress and prevented the decline of cognitive performance at middle age, but not at old age [37]. In the present work, we observed the beneficial effects of a daily moderate physical exercise program initiated at old age. Exercise improved learning and memory in different tests and also motor coordination (rotarod test), although it should be noted that the exercise program was performed with the rotarod and the animals may be more habituated to this device, and the benefit could also be related to the weight loss observed in these animals due to exercise. In line with these results, previous studies have indicated that exercise is able to improve brain function, also palliating age-related cognitive decline [38,39]. These changes have been associated both with vascular and metabolic improvements and with the possible capacity of exercise to directly induce structural and neurochemical changes in brain areas related to learning and memory [40,41]. Thus, exercise has been shown to increase angiogenesis and favor perfusion in the hippocampus [42], activate neurogenesis in the dentate gyrus [42] and increase synaptic plasticity [43,44], also increasing the complexity and number of dendritic spines [44,45,46,47], which promote memory function and protect against cognitive decline [48,49,50].

Many of the favorable effects of moderate exercise have been attributed to its ability to improve the antioxidant and redox status of the brain, attenuating the level of oxidative stress [37,51,52,53]. This beneficial effect of exercise could be responsible for the optimization of the activity of the limiting enzymes in the synthesis of monoamines TPH and TH in response to physical exercise, observed in the current work. In support of this assumption, it is known that these enzymes are very susceptible to oxidative damage [54,55,56], and different antioxidant treatments reverse the age-decreased activity of these enzymes [17,27,30]. The current results indicate that exercise was able to increase the levels of 5-HT, NA and DA, as a result of an increase in the activity of the above mentioned limiting enzymes, in the hippocampus, striatum and pineal gland, in agreement with previous studies that show changes in the central 5-HT, NA and DA levels after physical exercise [34,57,58].

SIRT1 expression decreased in the hippocampus throughout aging, and then exercise increased its levels. It is suggested that the increase in SIRT1 levels in the hippocampus, observed in this work, may be part of the mechanism of action of physical exercise on cognitive functions and brain monoamine levels. In this way, endurance exercise has been shown to induce expression of SIRT1 in different brain areas, including the cortex and hippocampus [59,60,61], but it did not change in other brain areas such as the cerebellum [61,62]. In contrast, in muscle the modulation of SIRT1 by exercise appears to be dependent on the type of exercise (for a further review, see Suwa and Sakuma [63]); moreover, a transient increase in SIRT1 protein followed by a prolonged decrease has been proposed [64]. Thus, activation of SIRT1 in the hippocampus through exercise may be beneficial against cognitive deficits, and could, at least in part, mediate some of the benefits of exercise observed in the present work.

The neurochemical results from the present work are in good correlation with the improving effect of exercise on memory and motor coordination observed. Thus, the improved motor coordination was closely related to the increased concentration of DA in the striatum [65,66]. It has also been described that the increased activity of the dopaminergic system in response to exercise can be associated with a raise in tyrosine hydroxylase activity through the activation of the calcium–calmodulin system [67]. Regarding the central noradrenergic system, it has been described that voluntary exercise increases NA levels in several regions of the brain [68] which may directly influence plasticity factors, facilitating sensory activation, focusing attention and enhancing learning during exercise [69]. Several studies have also suggested that exercise improves mood [2] and induces antidepressant effects [70,71] by modifying noradrenergic and serotonergic pathways. Thus, exercise appears to elevate tryptophan levels in the raphe nuclei [72] and also increases 5-HT release [73]. It has also been suggested that moderate exercise increases tryptophan levels in both the plasma and hippocampus [74,75], which may increase 5-HT production [70]. Increased plasma tryptophan levels as a consequence of exercise [74,75] could also increase 5-HT levels in the pineal gland, as observed in the present work, which may result in an improvement in melatonin levels [76]. It has been described that exercise can influence melatonin levels [77] and, consequently, improve the body’s circadian rhythms and the antioxidant properties of the hormone. The restoration of pineal function through exercise may have an additional beneficial effect on brain health, such as eliminating waste during deep sleep [78].

The possible role of RBAP46/48 in the protective effects of exercise was also investigated, and an age-associated reduction in hippocampal RBAP46/48 protein levels was corroborated by the current results, but exercise or catechin treatments were not able to reverse this reduction (see supplementary material Figure S2).

Although it has been described that some polyphenols such as epigallocatechin improve cardiac parameters [79], and resveratrol improved the effects of exercise in elderly rat hearts by enhancing FOXO3 phosphorylation via the synergetic activation of SIRT1 and PI3K/Akt signaling [80], we did not find potentiating effects of catechin over those induced by the exercise. In contrast, other research indicated that the flavanol epicatechin increased the effects of exercise on spatial memory [21]. It has also been described that exercise can potentiate the beneficial effects of the components of a healthy diet and vice versa, both at the cellular and molecular levels [81,82], exerting complementary actions by regulating brain energy metabolism [83] and modulating various signaling pathways linked to neuronal function and synaptic plasticity, affecting cognitive abilities [84]. On the contrary, the present results did not show an additional effect by catechin increasing the effects of exercise on cognitive tests or SIRT1 levels, despite the fact that catechin, chosen for this study, produced favorable effects on cognitive abilities and SIRT1 in a previous work [17]. Regarding these results, catechin alone did not reach the values achieved through exercise. It is likely that this effect depends on the intensity or duration of the program applied; in any case, it suggests that the changes induced by both therapies delay age-associated cognitive decline. However, an additive effect on brain monoamines after the application of exercise together with catechin has been observed in this study. Catechin, as previously described, also exerts a protective effect on TH and TPH [85]. Catechin is also able to activate, directly or indirectly, other mechanisms that may lead to the preservation of monoaminergic pathways impaired with age [85,86], restoring monoamine values. Thus, it is possible that the observed additive effect between exercise and catechin may be due to each treatment being able to activate or potentiate different mechanisms of action.

In summary, moderate exercise and the combined action of exercise and a polyphenol-enriched diet could be very useful as a therapy to delay or ameliorate the cognitive and motor decline associated with aging, by improving monoaminergic neurotransmitters and increasing SIRT1 levels in key cognitive regions. Therefore, a physically active lifestyle in combination with the intake of antioxidants may be one of the most effective and simple ways to maintain a healthy body and mind. For this reason, it seems necessary to promote the implementation of physical activity programs (determining the appropriate parameters of frequency, duration and intensity of exercise), combined with a diet rich in antioxidants that could alleviate the cognitive and motor deterioration associated with aging.

## Data Availability

Not applicable.

## Supplementary Materials

The following are available online at https://www.mdpi.com/article/10.3390/antiox10040621/s1. Figure S1: Effect on familiarization phase in the novel object recognition test in old rats, Figure S2: Effect of exercise and joint action of exercise with catechin antioxidant treatment on RbAp 48/46 (48 kDa) protein expression in the hippocampus of old rats.

## References

[1] Bishop N.A., Lu T., Yankner B.A. (2010). Neural mechanisms of ageing and cognitive decline. Nature.

[2] Basso J.L., Suzuki W.A. (2017). The effects of acute exercise on mood, cognition, neurophysiology, and neurochemical pathways: A Review. Brain Plast..

[3] Fernandes J., Arida R.M., Gomez-Pinilla F. (2017). Physical exercise as an epigenetic modulator of brain plasticity and cognition. Neurosci. Biobehav. Rev..

[4] Mandolesi L., Polverino A., Montuori S., Foti F., Ferraioli G., Sorrentino P., Sorrentino G. (2018). Effects of physical exercise on cognitive functioning and wellbeing: Biological and psychological benefits. Front. Psychol..

[5] Forster M.J., Dubey A., Dawson K.M., Stutts W.A., Lal H., Sohal R.S. (1996). Age-related losses of cognitive function and motor skills in mice are associated with oxidative protein damage in the brain. Proc. Natl. Acad. Sci. USA.

[6] Franceschi C., Bonafè M., Valensin S., Olivieri F., De Luca M., Ottaviani E., De Benedictis G. (2000). Inflamm-aging. An evolutionary perspective on immunosenescence. Ann. N. Y. Acad. Sci..

[7] De la Fuente M., Cruces J., Hernandez O., Ortega E. (2011). Strategies to improve the functions and redox state of the immune system in aged subjects. Curr. Pharm. Des..

[8] Di Benedetto S., Muller L., Wenger E., Duzel S., Pawelec G. (2017). Contribution of neuroinflammation and immunity to brain aging and the mitigating effects of physical and cognitive interventions. Neurosci. Biobehav. Rev..

[9] Suliman H.B., Piantadosi C.A. (2014). Mitochondrial biogenesis: Regulation by endogenous gases during inflammation and organ stress. Curr. Pharm. Des..

[10] Nogueiras R., Habegger K.M., Chaudhary N., Finan B., Banks A.S., Dietrich M.O., Horvath T.L., Sinclair D.A., Pfluger P.T., Tschöop M.H. (2012). Sirtuin 1 and Sirtuin 3: Physiological modulators of metabolism. Physiol. Rev..

[11] Ramis M.R., Esteban S., Miralles A., Tan D.X., Reiter R.J. (2015). Caloric restriction, resveratrol and melatonin: Role of SIRT1 and implications for aging and related-diseases. Mech. Ageing Dev..

[12] Pfluger P.T., Herranz D., Velasco-Miguel S., Serrano M., Tschöp M.H. (2008). Sirt1 protects against high-fat diet-induced metabolic damage. Proc. Natl. Acad. Sci. USA.

[13] Brunet A., Sweeney L.B., Sturgill J.F., Chua K.F., Greer P.L., Lin Y., Tran H., Ross S.E., Mostoslavsky R., Cohen H.Y. (2004). Stress-dependent regulation of FOXO transcription factors by the SIRT1 deacetylase. Science.

[14] Kobayashi Y., Furukawa-Hibi Y., Chen C., Horio Y., Isobe K., Ikeda K., Motoyama N. (2005). SIRT1 is critical regulator of FOXO-mediated transcription in response to oxidative stress. Int. J. Mol. Med..

[15] Herskovits A.Z., Guarente L. (2014). SIRT1 in neurodevelopment and brain senescence. Neuron.

[16] Sarubbo F., Esteban S., Miralles A., Moranta D. (2018). Effects of resveratrol and other polyphenols on Sirt1: Relevance to brain function during aging. Curr. Neuropharmacol..

[17] Ramis M.R., Sarubbo F., Tejada S., Jimenez M., Esteban S., Miralles A., Moranta D. (2020). Chronic Polyphenon-60 or Catechin treatments increase brain monoamines syntheses and hippocampal SIRT1 levels improving cognition in aged rats. Nutrients.

[18] Kosmidis S., Polyzos A., Harvey L., Youssef M., Denny C.A., Dranovsky A., Kandel E.R. (2018). RbAp48 protein is a critical component of GPR158/OCN signaling and ameliorates age-related memory loss. Cell Rep..

[19] Pavlopoulos E., Jones S., Kosmidis S., Close M., Kim C., Kovalerchik O., Small S.A., Kandel E.R. (2013). Molecular mechanism for age-related memory loss: The histone-binding protein RbAp48. Sci. Transl. Med..

[20] Anderton B.H. (2002). Ageing of the brain. Mech. Ageing Dev..

[21] Van Praag H. (2009). Exercise and the brain: Something to chew on. Trends Neurosci..

[22] Esteban S., Garau C., Aparicio S., Moranta D., Barcelo P., Fiol M.A., Rial R. (2010). Chronic melatonin treatment and its precursor L-tryptophan improve the monoaminergic neurotransmission and related behavior in the aged rat brain. J. Pineal Res..

[23] Sharma S., Rakoczy S., Brown-Borg H. (2010). Assessment of spatial memory in mice. Life Sci..

[24] Barrett G.L., Bennie A., Trieu J., Ping S., Tsafoulis C. (2009). The chronology of age-related spatial learning impairment in two rat strains, as tested by the Barnes maze. Behav. Neurosci..

[25] Rueda-Orozco P., Soria-Gomez E., Montes-Rodriguez C., Martínez-Vargas M., Galicia O., Navarro L., Prospero-García O. (2008). A potential function of endocannabinoids in the selection of a navigation strategy by rats. Psychopharmacology.

[26] Antunes M., Biala G. (2012). The novel object recognition memory: Neurobiology, test procedure, and its modifications. Cogn. Process..

[27] Ramis M.R., Sarubbo F., Terrasa J.L., Moranta D., Aparicio S., Miralles A., Esteban S. (2016). Chronic α-tocopherol increases central monoamines synthesis and improves cognitive and motor abilities in old rats. Rejuvenation Res..

[28] Walther D., Peter J., Bashammakh S., Hörtnagl H., Voits M., Fink H., Bader M. (2003). Synthesis of serotonin by a second tryptophan hydroxylase isoform. Science.

[29] Zhang X., Beaulieu J.M., Sotnikova T.D., Gainetdinov R.R., Caron M.G. (2004). Tryptophan hydroxylase-2 controls brain serotonin synthesis. Science.

[30] Ramis M.R., Sarubbo F., Moranta D., Tejada S., Lladó J., Miralles A., Esteban S. (2021). Cognitive and neurochemical changes following polyphenol-enriched diet in rats. Nutrients.

[31] Moranta D., Barcelo P., Aparicio S., Garau C., Sarubbo F., Ramis M., Nicolau C., Esteban S. (2014). Intake of melatonin increases tryptophan hydroxylase type 1 activity in aged rats: Preliminary study. Exp. Gerontol..

[32] Norton S., Matthews F.E., Barnes D.E., Yaffe K., Brayne C. (2014). Potential for primary prevention of Alzheimer’s disease: An analysis of population-based data. Lancet Neurol..

[33] Ahlskog J.E., Geda Y.E., Graff-Radford N.R., Petersen R.C. (2011). Physical exercise as a preventive or disease modifying treatment of dementia and brain aging. Mayo Clin. Proc..

[34] Ma Q. (2008). Beneficial effects of moderate voluntary physical exercise and its biological mechanisms on brain health. Neurosci. Bull..

[35] Ngandu T., Lehtisalo J., Solomon A., Levälahti E., Ahtiluoto S., Antikainen R., Bäckman L., Hänninen T., Jula A., Laatikainen T. (2015). A 2 year multidomain intervention of diet, exercise, cognitive training, and vascular risk monitoring versus control to prevent cognitive decline in at-risk elderly people (FINGER): A randomised controlled trial. Lancet.

[36] Prakash R.S., Voss M.W., Erickson K.I., Kramer A.F. (2015). Physical activity and cognitive vitality. Ann. Rev. Psychol..

[37] Navarro A., Gomez C., López-Cepero J.M., Boveris A. (2004). Beneficial effects of moderate exercise on mice aging: Survival, behavior, oxidative stress, and mitochondrial electron transfer. Am. J. Physiol. Regul. Integr. Comp. Physiol..

[38] Bronner L.L., Kanter D.S., Manson J.E. (1995). Primary prevention of stroke. N. Engl. J. Med..

[39] Oliff H.S., Berchtold N.C., Isackson P., Cotman C.W. (1998). Exercise-induced regulation of brain-derived neurotrophic factor (BDNF) transcripts in the rat hippocampus. Brain Res. Mol. Brain Res..

[40] Duzel E., van Praag H., Sendtner M. (2016). Can physical exercise in old age improve memory and hippocampal function?. Brain.

[41] Erickson K.I., Voss M.W., Prakash R.S., Basak C., Szabo A., Chaddock L., Kim J.S., Heo S., Alves H., White S.M. (2011). Exercise training increases size of hippocampus and improves memory. Proc. Natl. Acad. Sci. USA.

[42] Pereira A.C., Huddleston D.E., Brickman A.M., Sosunov A.A., Hen R., McKhann G.M., Sloan R., Gage F.H., Brown T.R., Small S.A. (2007). An in vivo correlate of exercise-induced neurogenesis in the adult dentate gyrus. Proc. Natl. Acad. Sci. USA.

[43] Farmer J., Zhao X., van Praag H., Wodtke K., Gage F.H., and Christie B.R. (2004). Effects of voluntary exercise on synaptic plasticity and gene expression in the dentate gyrus of adult male Sprague-Dawley rats in vivo. Neuroscience.

[44] Vivar C., Potter M.C., van Praag H. (2012). All about running: Synaptic plasticity, growth factors and adult hippocampal neurogenesis. Neurogenesis Neural Plast..

[45] Eadie B.D., Redila V.A., Christie B.R. (2005). Voluntary exercise alters the cytoarchitecture of the adult dentate gyrus by increasing cellular proliferation, dendritic complexity, and spine density. J. Comp. Neurol..

[46] Siette J., Westbrook R.F., Cotman C., Sidhu K., Zhu W., Sachdev P., Valenzuela M.J. (2013). Age-specific effects of voluntary exercise on memory and the older brain. Biol. Psychiatry.

[47] Stranahan A.M., Khalil D., Gould E. (2007). Running induces widespread structural alterations in the hippocampus and entorhinal cortex. Hippocampus.

[48] Cotman C.W., Engesser-Cesar C. (2002). Exercise enhances and protects brain function. Exerc. Sport Sci. Rev..

[49] Van Praag H., Christie B.R., Sejnowski T.J., Gage F.H. (1999). Running enhances neurogenesis, learning, and LTP in mice. Proc. Natl. Acad. Sci. USA.

[50] Van Praag H., Kempermann G., Gage F.H. (1999). Running increases cell proliferation and neurogenesis in the adult mouse dentate gyrus. Nat. Neurosci..

[51] Franzoni F., Federighi G., Fusi J., Agosta V., Cerri E., Banducci R., Petrocchi A., Bernardi R., Innocenti A., Pruneti C. (2017). Physical exercise improves total antioxidant capacity and gene expression in rat hippocampal tissue. Arch. Ital. Biol..

[52] Sallam N., Laher I. (2016). Exercise modulates oxidative stress and inflammation in aging and cardiovascular diseases. Oxid. Med. Cell. Longev..

[53] Somani S.M., Ravi R., Rybak L.P. (1995). Effect of exercise training on antioxidant system in brain regions of rat. Pharm. Biochem. Behav..

[54] Cash C.D. (1998). Why tryptophan hydroxylase is difficult to purify: A reactive oxygen-derived species-mediated phenomenon that may be implicated in human pathology. Gen. Pharmacol..

[55] De La Cruz C.P., Revilla E., Venero J.L., Ayala A., Cano J., Machado A. (1996). Oxidative inactivation of tyrosine hydroxylase in substantia nigra of aged rat. Free Radic. Biol. Med..

[56] Hussain A., Mitra A. (2000). Effect of aging on tryptophan hydroxylase in rat brain: Implications on serotonin level. Drug Metab. Dispos..

[57] Meeusen R., De Meirleir K. (1995). Exercise and brain neurotransmission. Sports Med..

[58] Min Y.K., Chung S.H., Lee J.S., Kim S.S., Shin H.D., Lim B.V., Shin M.C., Jang M.H., Kim E.H., Kim C.J. (2003). Red ginseng inhibits exercise induced increase in 5-hydroxytryptamine synthesis and tryptophan hydroxylase expression in dorsal raphe of rats. J. Pharmacol. Sci..

[59] Bayod S., Guzmán-Brambila C., Sanchez-Roige S., Lalanza J.F., Kaliman P., Ortuño-Sahagun D., Escorihuela R.M., Pallàs M. (2015). Voluntary exercise promotes beneficial anti-aging mechanisms in SAMP8 female brain. J. Mol. Neurosci..

[60] Bayod S., Del Valle J., Canudas A.M., Lalanza J.F., Sanchez-Roige S., Camins A., Escorihuela R.M., Pallàs M. (2011). Long-term treadmill exercise induces neuroprotective molecular changes in rat brain. J. Appl. Physiol..

[61] Steiner J.L., Murphy E.A., McClellan J.L., Carmichael M.D., Davis J.M. (2011). Exercise training increases mitochondrial biogenesis in the brain. J. Appl. Physiol..

[62] Marton O., Koltai E., Nyakas C., Bakonyi T., Zenteno-Savin T., Kumagai S., Goto S., Radak Z. (2010). Aging and exercise affect the level of protein acetylation and SIRT1 activity in cerebellum of male rats. Biogerontology.

[63] Suwa M., Sakuma K. (2013). The potential role of sirtuins regarding the effects of exercise on aging-related diseases. Curr. Aging Sci..

[64] Gurd B.J., Yoshida Y., McFarlan J.T., Holloway G.P., Moyes C.D., Heigenhauser G.J.F., Spriet L., Bonen A. (2011). Nuclear SIRT1 activity, but not protein content, regulates mitochondrial biogenesis in rat and human skeletal muscle. Am. J. Physiol. Regul. Integr. Comp. Physiol..

[65] Kravitz A.V., Kreitzer A.C. (2012). Striatal mechanisms underlying movement, reinforcement, and punishment. Physiology.

[66] Rabelo P.C., Almeida T.F., Guimaraes J.B., Barcellos L.A., Cordeiro L.M., Moraes M.M., Coimbra C.C., Szawka R.E., Soares D.D. (2015). Intrinsic exercise capacity is related to differential monoaminergic activity in the rat forebrain. Brain Res. Bull..

[67] Sutoo D., Akiyama K. (2003). Regulation of brain function by exercise. Neurobiol. Dis..

[68] Dunn A.L., Reigle T.G., Youngstedt S.D., Armstrong R.B., Dishman R.K. (1996). Brain norepinephrine and metabolites after treadmill training and wheel running in rats. Med. Sci. Sports Exerc..

[69] Nicastro T.M., Greenwood B.N. (2016). Central monoaminergic systems are a site of convergence of signals conveying the experience of exercise to brain circuits involved in cognition and emotional behavior. Curr. Zool..

[70] Ernst C., Olson A.K., Pinel J.P., Lam R.W., Christie B.R. (2006). Antidepressant effects of exercise: Evidence for an adult-neurogenesis hypothesis?. J. Psychiatry Neurosci..

[71] Ota K.T., Duman R.S. (2013). Environmental and pharmacological modulations of cellular plasticity: Role in the pathophysiology and treatment of depression. Neurobiol. Dis..

[72] Lim B.V., Jang M.H., Shin M.C., Kim H.B., Kim Y.J., Kim Y.P., Chung J.H., Kim H., Shin M.S., Kim S.S. (2001). Caffeine inhibits exercise induced increase in tryptophan hydroxylase expression in dorsal and median raphe of Sprague-Dawley rats. Neurosci. Lett..

[73] Xu C.X., Liu H.T., Wang J. (2008). Changes of 5-hydroxytryptamine and tryptophan hydroxylase expression in the ventral horn of spinal cord. Neurosci. Bull..

[74] Chaouloff F., Kennett G.A., Serrurrier B., Merino D., Curzon G. (1986). Amino acid analysis demonstrates that increased plasma free tryptophan causes the increase of brain tryptophan during exercise in the rat. J. Neurochem..

[75] Chaouloff F., Laude D., Elghozi J.L. (1989). Physical exercise: Evidence for differential consequences of tryptophan on 5-HT synthesis and metabolism in central serotonergic cell bodies and terminals. J. Neural Transm..

[76] Esteban S., Nicolau C., Garmundi A., Rial R.V., Rodríguez A.B., Ortega E., Ibars C.B. (2004). Effect of orally administered L-tryptophan on serotonin, melatonin and the innate immune response. Mol. Cell. Biochem..

[77] Escames G., Ozturk G., Baño-Otálora B., Pozo M.J., Madrid J.A., Reiter R.J., Serrano E., Concepción M., Acuña-Castroviejo D. (2012). Exercise and melatonin in humans: Reciprocal benefits. J. Pineal Res..

[78] Van Alphen B., Evan R., Semenza E.R., Yap M., van Swinderen B., Allada R. (2021). A deep sleep stage in Drosophila with a functional role in waste clearance. Sci. Adv..

[79] Reiter C.E.N., Kim J., Quon M.J. (2010). Green tea polyphenol epigallocatechin gallate reduces endothelin-1 expression and secretion in vascular endothelial cells: Roles for AMP-activated protein kinase, Akt, and FOXO1. Endocrinology.

[80] Lin C.H., Lin C.C., Ting W.J., Pai P.Y., Kuo C.H., Ho T.J., Kuo W.W., Chang C.H., Huang C.Y., Lin W.T. (2014). Resveratrol enhanced FOXO3 phosphorylation via synergetic activation of SIRT1 and PI3K/Akt signaling to improve the effects of exercise in elderly rat hearts. Age.

[81] Murphy T., Dias G.P., Thuret S. (2014). Effects of diet on brain plasticity in animal and human studies: Mind the gap. Neural Plast..

[82] Van Praag H., Lucero M.J., Yeo G.W., Stecker K., Heivand N., Zhao C., Yip E., Afanador M., Schroeter H., Hammerstone J. (2007). Plant-derived flavanol (-)epicatechin enhances angiogenesis and retention of spatial memory in mice. J. Neurosci..

[83] Gomez-Pinilla F., Tyagi E. (2013). Diet and cognition: Interplay between cell metabolism and neuronal plasticity. Curr. Opin. Clin. Nutr. Metab. Care.

[84] Gomez-Pinilla F., Nguyen T. (2012). Natural mood foods: The actions of polyphenols against psychiatric and cognitive disorders. Nutr. Neurosci..

[85] Rai A., Gill M., Kinra M., Shetty R., Krishnadas N., Rao C.M., Sumalatha S., Kumar N. (2019). Catechin ameliorates depressive symptoms in Sprague Dawley rats subjected to chronic unpredictable mild stress by decreasing oxidative stress. Biomed. Rep..

[86] Acosta S., Jernberg J., Sanberg C.D., Sanberg P.R., Small B.J., Gemma C., Bickford P.C. (2010). NT-020, a natural therapeutic approach to optimize spatial memory performance and increase neural progenitor cell proliferation and decrease inflammation in the aged rat. Rejuvenation Res..

