# Supplementary material for: Neurochemical and Cognitive Beneficial Effects of Moderate Physical Activity and Catechin in Aged Rats
Source: Antioxidants (Basel). 2021 Apr 19;10(4):621. doi: 10.3390/antiox10040621 (PMC8072822; doi:10.3390/antiox10040621)
Supplement: Supplementary file 1 [file antioxidants-10-00621-s001.zip › antioxidants-1160460-supplementary.pdf]

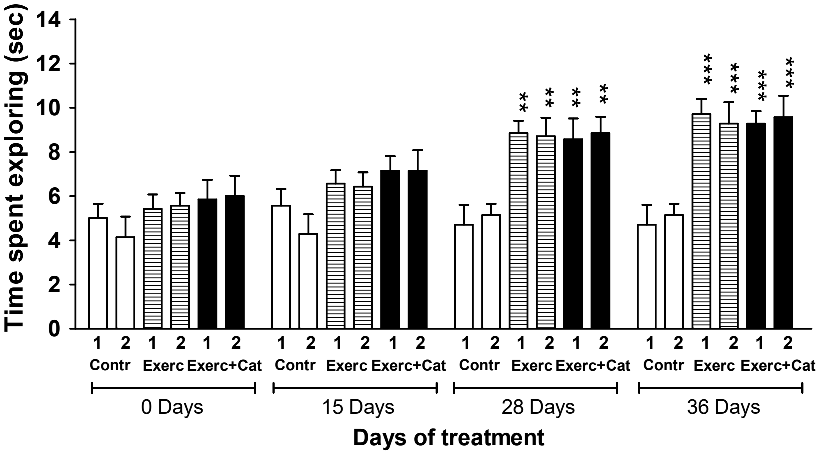

Figure S1. Effect of exercise and the combined action of exercise and catechin treatment on familiarization phase in the novel object recognition test in old rats. Bars represent the mean ± SEM of the total time spent exploring each object (1, left object; 2, right object) of animals subjected to exercise (Exerc, $n = 7$) and the exercise plus catechin treatment group (Exerc + Cat, $n = 7$) respect to the control group ($n = 7$). $^{**}p < 0.01$, $^{***}p < 0.001$ when compared to the control group.

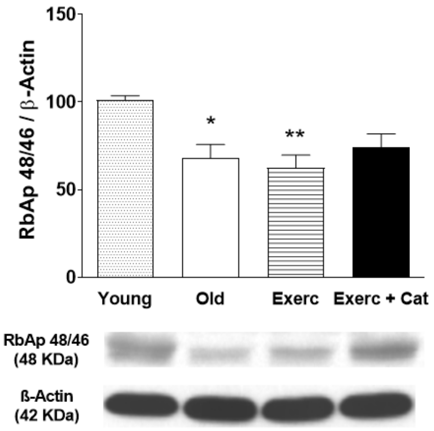

Figure S2. Effect of exercise and the combined action of exercise and catechin antioxidant treatment on RbAp 48/46 (48 kDa) protein expression in hippocampus of old rats. Bars represent mean ± SEM of protein levels per group (animals subjected to exercise, Exerc, n=7; exercise together with catechin, Exerc + Cat, n=7; old control group, Old, n=6) expressed as percentage relative to the young group (n=6). Protein level was normalized to β-actin content and each sample was analyzed in three different membranes. One-way ANOVA detected a significant effect ($F(3,22)=6.052$, $p=0.0036$). $^{*}p<0.05$, $^{**}p<0.01$ when compared with young animals in the control group (one-way ANOVA, followed by Bonferroni test). A representative immunoblot of the changes obtained in RbAp 48/46, with no changes in ß-actin (loading pattern), is shown below graph.
